# Development of New Bio-Composite of PEO/Silk Fibroin Blends Loaded with Piezoelectric Material

**DOI:** 10.3390/polym14194209

**Published:** 2022-10-07

**Authors:** Hassan Fouad, Khalil Abdelrazek Khalil, Basheer A. Alshammari, Abdalla Abdal-hay, Nasser M. Abd El-salam

**Affiliations:** 1 Applied Medical Science Department, Community College, King Saud University, Riyadh 11433, Saudi Arabia; 2 Department of Mechanical and Nuclear Engineering, College of Engineering, University of Sharjah, Sharjah P.O. Box 27272, United Arab Emirates; 3 Materials Science Research Institute, King Abdulaziz City for Science and Technology, Riyadh 11442, Saudi Arabia; 4 School of Dentistry, Herston Campus, University of Queensland, St Lucia, QLD 4072, Australia; 5 Department of Engineering Materials and Mechanical Design, Faculty of Engineering, South Valley University, Qena 83523, Egypt

**Keywords:** biocomposites, PEO/silk, piezoelectric material, electrospinning, CTE

## Abstract

New bio-composite nanofibers composed of polyethylene oxide (PEO)/silk fibroin (SF)/barium titanate (BaTiO_3_) are introduced in this study. The SF solution was added to the PEO solution to form a PEO/SF blend with different weight percentages (5, 10, 15, 20 wt.%). The PEO/15 wt.% SF blend was selected to continue the experimental plan based on the optimum nanofiber morphology. Different wt.% of BaTiO_3_ particles (0.2, 0.4, 0.8, 1 wt.%) were added to the PEO/15 wt.% SF blend solution, and the suspensions obtained were introduced to an electrospinning device. The fabricated tissue was characterized by scanning electron microscope (SEM), X-ray diffraction (XRD) and Fourier-transform infrared (FTIR) spectroscopy. The zeta potential of the solution and the piezoelectric performance of the fabricated tissue were characterized. A newly designed pizoTester was used to investigate piezoelectric properties. The results showed that a well-organized, smooth PEO/15 wt.% SF/0.2 wt.% BaTiO_3_ nanofiber composite with low bead contents was obtained. Improved properties and electrical coupling were achieved in the newly introduced material. Electrospun PEO/15 wt.% SF/0.2 wt.% BaTiO_3_ mats increased the output voltage (1150 mV) compared to pristine PEO and PEO/SF composite fibers (410 and 290 mV, respectively) upon applying 20 N force at 5 Hz frequency. The observed enhancement in piezoelectric properties suggests that the prepared composite could be a promising material in cardiac tissue engineering (CTE).

## 1. Introduction

Cardiac tissue engineering (CTE) is one of the promising approaches and novel strategies to prevent and fully treat heart loss/blighter at a relatively low cost. An ideal scaffold for cardiac tissue engineering must be electrically conductive, mechanically stable, biocompatible, and topographically suitable.

These CTE bio-composites are formed using plant or animal-based natural fibers and biodegradable polymer matrices [1,2,3,4,5,6,7,8]. Some significant development has been made in CTE to utilize electroactive materials. However, they are still far from real clinical applications. The natural fibers enhance the resultant composite’s physical, mechanical, and biological properties of the polymer matrix. They have low production costs, low density, biodegradability, non-toxic behavior, high stiffness, and specific strength. As a matrix of CTE biomaterials, PEO has an extensive range of erosive degradation/absorption, but its biocompatibility is relatively low. It is an attractive polymer that can form complexes with many small molecules [9,10,11,12,13].

Moreover, PEO is widely available with a range of molecular weights. It exhibits exciting non-toxicity, biodegradability, biocompatibility, and solubility in aqueous solutions. Due to these properties, PEO is used in a broad range of applications [14,15,16,17,18,19,20].

In contrast, Silk fibroin-based natural material exhibits high biocompatibility and low immunogenicity [21,22,23,24,25,26,27]. SF’s primary structure consists of a repeating pattern of glycine-alanine-glycine-alanine-glycine-serine amino acids. These amino acids undergo anti-parallel self-assembly of β-sheet structures. β-sheets are cross-linked using Van der Waals interactions and hydrogen bonding, which are responsible for the mechanical properties of SF fibers [28,29,30,31,32,33,34,35,36,37,38,39]. SF solutions are efficiently electrospun, but the concentration required for satisfactory viscosity to form fibers is considerably high [40,41,42,43]. These stability issues result from SF self-assembly to form insoluble β-sheets, which are not able to spin [44,45]. The standard practice/strategy is to add the SF solutions into a polymer matrix such as PEO to spin the solutions. We assume that a controlled blend of PEO and SF can produce novel composite scaffold materials with unique electrical, chemical, and biological characteristics.

Moreover, integrating nanoparticle features and electrical stimulation (via piezoelectric conductive materials) has promoted cardiac tissue repair and regeneration. Such nanomaterials will improve cardiac cell behavior, such as proliferation, migration, and cardio-myogenic differentiation in stem cells [46,47]. In this regard, BaTiO_3_ nanoparticles have been used to enhance the piezoelectric performance of PEO/SF biocomposites.

On the other hand, the electrospinning technique has been used to form fine nonwoven fibers as a 3D scaffold for tissue engineering (TE). Using electrospun nanofibers for TE not only helps in improving the interactions and functions of cells on the surface, but also allows for the diffusion of nutrients and metabolic waste between the scaffold and cells [48,49,50]. These applications require a high surface area of fibers. At the same time, the thickness of the final mesh can be controlled through electrospinning duration. Usually, a dry or wet spinning process produces fibers from the protein solution. However, electrospinning is more capable of generating nano/microfibers. [51,52].

As far as we know, there is no study published that deals with such composites supported by piezoelectric materials for medical applications. Thus, the present study aims to develop and fabricate a new bio-composite with enhanced piezoelectricity properties using biodegradable scaffolds of PEO/SF mats supported with BaTiO_3_ as piezoelectric materials. We believe that these biodegradable scaffolds will provide unique electrical integration functions to the scaffolds.

## 2. Materials and Methods

### 2.1. Preparation of the Blends

Silk fibroin (SF), a 5% solution product from an advanced biomatrix, was used as received. The SF was stored at −70 °C until ready to use. Before mixing with PEO, the SF was thawed overnight in the refrigerator until the solution became clear with relatively low viscosity. A polyethylene oxide (PEO, MW 100,000, Sigma Aldrich, St. Louis, MI, USA) powder was used as received. The amount of PEO used to prepare the solution was 12 wt.% dissolved in 88 wt.% of solvents composed of 80 wt.% of DI (deionized) water and 20 wt.% of ethanol. The beaker was then sealed and kept at 80 °C while constantly stirring at 300 RPM until a uniform solution density was obtained.

**Figure 1:**
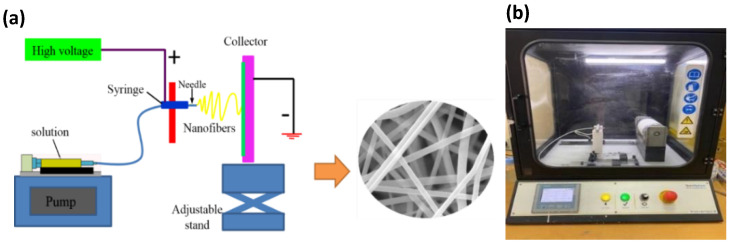
(**a**) schematic diagram of the electrospinning approaches, (**b**) NanoSpinner device.

Finally, the SF solution was added to the PEO solution to form a PEO/SF blend with different weight percentages (5, 10, 15, 20 wt.%). Based on the optimum nanofiber morphology, the PEO/15 wt.% SF blends were selected to continue the experimental plan. BaTiO_3_ particles were purchased by Nanostructured & Amorphous Materials, Inc. (Houston, TX, USA), with an average size of 100 nm. Different wt.% of BaTiO_3_ particles (0.2, 0.4, 0.8, 1 wt.%) were added to the PEO/15 wt.% silk fibroin blends solution. The obtained suspensions were introduced into an electrospinning device (Nanospinner, Inovenso, Turkey) at 20 kV. A syringe with a 0.50 mm inner diameter needle tip was used. The needle was placed 14 cm away from the collecting drum and wrapped with aluminum foil. The electrospinning set-up is shown in Figure 1. The same conditions (applying voltage: 20 kV, feed rate: 0.3 mL/h, the distance between tip and collector: 14 cm, drum speed: 250 RPM) were used for all samples.

### 2.2. Characterization

#### 2.2.1. Zeta Potential

To evaluate the zeta potential of the solution before electrospinning, batch experiments were carried out by using 10 mg of the solution at an initial pH7 to determine the charge of the material at an electric double layer of surfaces in an aqueous solution using an Omega Z cuvette with a particle analyzer (LitesizerTM 500).

#### 2.2.2. XRD, SEM, and FTIR Evaluation

The nanofibers produced were characterized by conventional Bragg–Brentano X-ray diffraction (XRD) to evaluate the structure of the nanofibers. XRD was conducted to analyze the phase and crystallinity using a Rigaku X-ray diffractometer (Rigaku Co., Tokyo, Japan) with Cu Kα (λ = 1.54056 Å). The study used 2θ angles from 5° to 80°. A spectrometer (JY H800UV) equipped with an optical microscope was used to collect Raman spectra at room temperature with a radiation wavelength of 532 nm. Further, the (FE-SEM) field-emission scanning electron microscope (FE-SEM, Hitachi S-7400, Ibaraki, Japan) was employed to study the fabricated biocomposites’ structural features and chemical composition. All samples were coated with a platinum conductive material to prevent charge buildup on the specimen surface. The coating was thick enough to avoid charging but not thick enough to obscure specimen surface details. FTIR spectroscopy of the prepared samples was registered at a reflection mode by a model Thermo Nicolet 6700 Fourier transform IR spectrometer, in the range of 500–3000 cm^−1^ and with an accuracy of ±0.09 cm^−1^. The KBr disc method was used.

#### 2.2.3. Piezoelectric Characteristics

**Figure 2:**
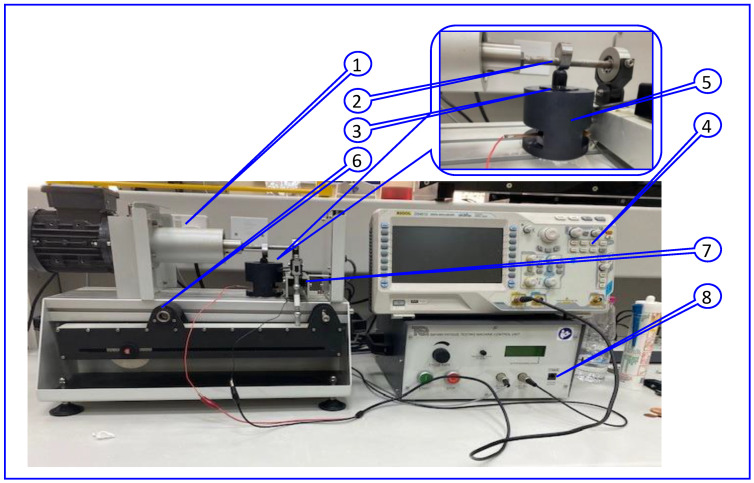
Picture of assembled setup during the examination of the piezoelectric test: (1) a TQ SM1090 fatigue test machine, (2) a camshaft, (3) vertical tapping shaft, (4) oscilloscope, (5) container, (6) force adjustable arm, (7) load cell, (8) frequency and force monitoring.

A PiezoTester device designed and manufactured in our lab was used to evaluate the piezoelectric properties. A SolidWorks drawing is provided in the Figure S1: SolidWorks. The tester was developed based on the concept of Sorayani et al. [51]. A live video explaining the detailed mechanism is provided in Video S1: PiezoTester mechanism. Using different forces and frequencies, the samples were evaluated in tapping mode (dynamic load). Figure 2 shows a picture of the assembled setup during the examination. The system is comprised of the following parts: a TQ SM1090 fatigue test machine (part 1) with an electric motor that generates rotary motion with different cycles/second cycles was used. A sample of the fatigue test machine was replaced by a camshaft (part 2) that converts the rotary motion into a reciprocating motion using a vertical tapping shaft (part 3). The sample was fixed between two highly conductive copper discs connected to an oscilloscope (part 4) and placed on a container (part 5) under the vertical tapping shaft (part 3).

Force was applied using an adjustable arm (part 6) connected to a load cell (part 7). Three different samples, mainly PEO, PEO/15 wt.% SF, and PEO/15 wt.% SF/0.2 wt.% BaTiO_3_, were tested using this system. A 500 μm-measured thickness of the electrospun samples was used for this experiment. The PizoTester device applies the specific dynamic force of different magnitudes with different frequencies to the prepared sample. A RIGOL DS4012 digital Oscilloscope then measures that output piezoelectric voltage. Further, the output voltage of the load cell shows the amount and frequency of applying an alternative force (part 8 in Figure 2).

## 3. Results and Discussion

### 3.1. Zeta Potential and Fiber Morphology

**Figure 3:**
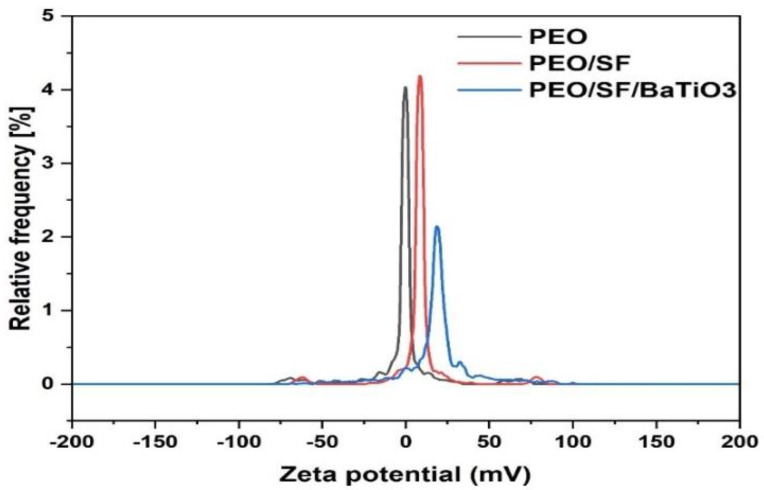
Zeta−potential distribution and dielectric displacement data for PEO pure, PEO/SF, and SF/PEO/BaTiO_3_.

Zeta potential is one of the most critical parameters in defining the physicochemical properties of a colloidal particle. A zeta potential test was conducted to optimize the electrospinning solution’s formulations and predict interactions between different constituents. It was also necessary as an aid in predicting long-term stability. Zeta-potential distribution analyses are reported in Figure 3. The zeta potential for pristine PEO, PEO/SF, and PEO/SF/BaTiO_3_ are shown from left to right, respectively. As the figure suggests, only a tiny fraction of PEO appears to carry a zero zeta potential, and the majority have a positive zeta potential. When SF is mixed in a binary system with the pure PEO material, which has different zeta-potential distributions, the resulting zeta-potential distribution was slightly shifted and positively changed, confirming the absence of interactions between the two biopolymers. A schematic demonstration of how the zeta-potential distribution analysis technique can explain the lack of interaction in the trinary mixture (PEO-SF-BaTiO_3_) is shown in the diagram to the right in Figure 3. The value of the zeta potential is also reported in Figure 3. As can be seen in this figure, it is clear that the zeta-potential distribution of pristine PEO has a lower value than the binary PEO/SF system. With the presence of BaTiO_3_, the value decreased dramatically. However, the PEO-SF-BaTiO_3_ remained well-suspended within the system due to specific repulsive mechanisms.

Moreover, negative zeta potentials at the PEO-SF-BaTiO_3_ composites were observed due to BaTiO_3_ fine particles. BaTiO3 particles can be characterized by a broad negative zeta-potential values distribution. Overall, the width of the corresponding zeta-potential distributions initially decreased with the addition of SF, then increased almost twofold in the presence of SF-BaTiO_3_. The maximum width on the curve roughly coincides with the PEO-SF-BaTiO_3_ sample. We can conclude that changes in the width of the zeta-potential distributions of the PEO-SF-BaTiO_3_ composites were attributed to the addition of the piezoelectric fine particles, confirming the absence of interactions between the biocomposite components.

**Figure 4:**
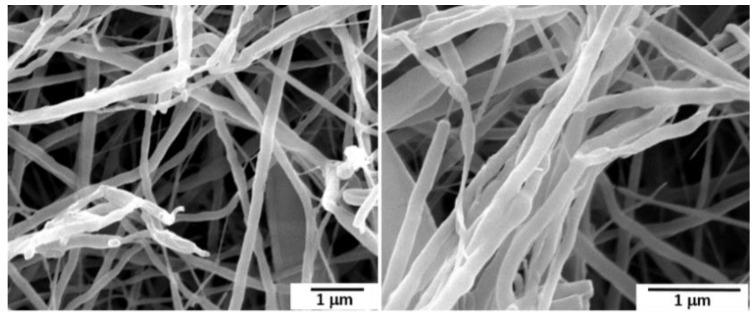
SEM micrograph of the pristine PEO nanofibers at different magnifications.

Figure 4 provides SEM micrographs of the pure PEO nanofibers at different magnifications. The results showed that all obtained fibers were randomly oriented, and the diameters were in the nanoscale range. Some nanofibers have a stable and smooth surface, with the narrowest diameter distribution. The fibers’ diameter distribution slightly broadened, with a maximum centered at 500 nm. Blobs with a thickening of the mean diameter in certain areas have been observed, confirmed by many interconnections among the fibers. The phenomenon is common and usually occurs if the addition of the fibers into a unique structure occurs before solvent removal and solidification of the polymeric structure [52].

**Figure 5:**
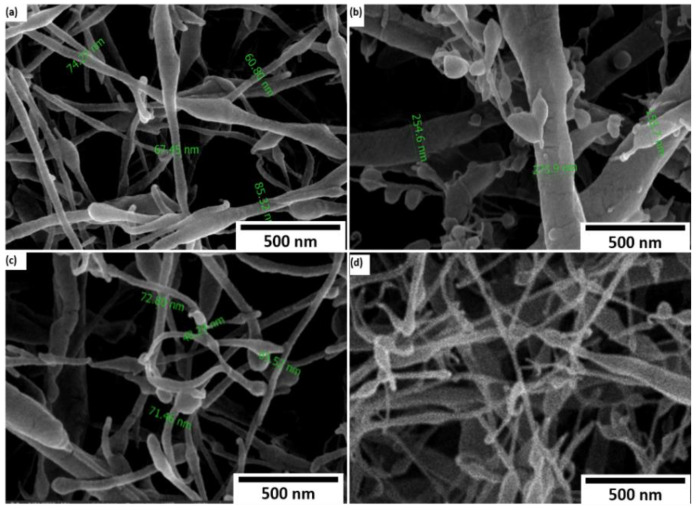
PEO/SF nanofibers with different SF wt.%: (**a**) 5 wt.%, (**b**) 10 wt.%, (**c**) 15 wt.%, and (**d**) 20 wt.%.

Figure 5 shows the obtained morphology of the electrospun PEO/SF nanofiber mixture with different SF weight percentages ((a) 5 wt.%, (b) 10 wt.%, (c) 15 wt.%, and (d) 20 wt.%) of the nanofibers after spinning. After electrospinning, all samples showed similar fiber types with a somewhat porous structure and casual dispersion. Moreover, Figure 5a shows that PEO/5 wt.% SF fibers were rough, solid, and narrow strips of fabric that did not form beads. However, the PEO/10 wt.% SF (Figure 5b) and PEO/15 wt.% SF (Figure 5c) fibers were denser, coarser, and more intensive than pure PEO with low-SF-concentration nanofibers.

Visible beads were formed during the spinning process, and the fiber diameter decreased from around 270 nm to 72 nm. As for PEO/20 wt.% SF nanofibers, the beads dramatically increase in Figure 5d. A fracture of the fibrous structure within a single nanofiber was observed. It can be concluded that PEO exhibited excellent fiber-forming ability, whereas the addition of SF affected its morphology based on the SEM results. A survey morphology of samples shows the presence of electro-spraying phenomena, depending on the presence of additives. The results recommend that PEO/15 wt.% SF fibers are the optimum concentration for the amount of SF that can be added to the PEO, as the nanofibers in this design were denser and more intensive than pure PEO with low SF concentration nanofibers. Moreover, adding more than 15% SF will result in poor morphology with many beads.

**Figure 6:**
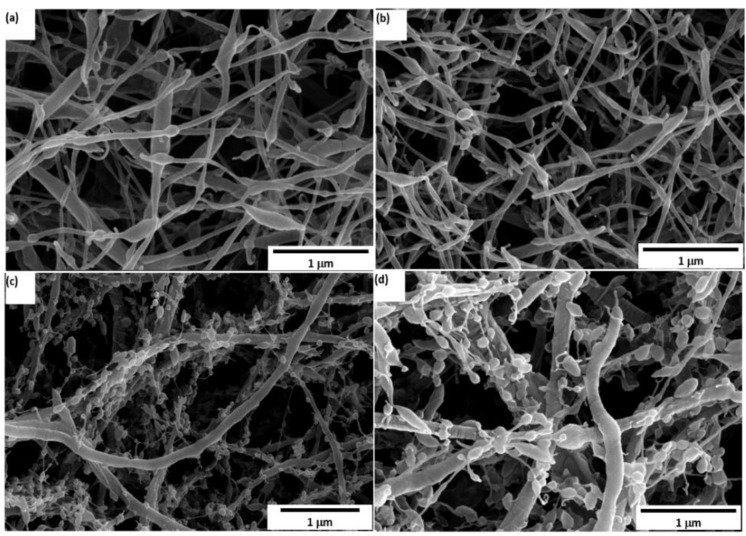
PEO/15 wt.% SF/BaTiO_3_ with different BaTiO_3_ content: (**a**) 0.2 wt.%, (**b**) 0.4 wt.%, (**c**) 0.8 wt.%, and (**d**) 1 wt.%.

Figure 6 shows the morphology of the PEO/15 wt.% SF nanofiber mixture produced with different BaTiO_3_ wt.% ((a) 0.2 wt.%, (b) 0.4 wt.%, (c) 0.8 wt.%, and (d) 1.0 wt.%) of nanofibers after spinning. Almost similar fiber morphology was produced. Some beads were found in the fibers with random dispersion and linear stretching after electrospinning. Moreover, Figure 6a reveals that PEO/15 wt.% SF with a lower percentage of BaTiO_3_ particles was somewhat smooth with soft bead content. However, a precise amount of beads was accumulated when the BaTiO_3_ wt.% increased (Figure 6b–d). As for PEO/15 wt.% SF with 1 wt.% BaTiO_3_ content nanofibers, as shown in Figure 5d, the beads are dramatically increased, and a crack in the fibrous structure between a single nanofiber was presented. Moreover, the diameters of the nanofibers were reduced from 200 nm to around 70 nm as the BaTiO_3_ content increased. These results suggested that low contents of BaTiO_3_ will be more efficient in terms of fiber morphology.

### 3.2. FTIR, XRD, and Raman Characterizations

**Figure 7:**
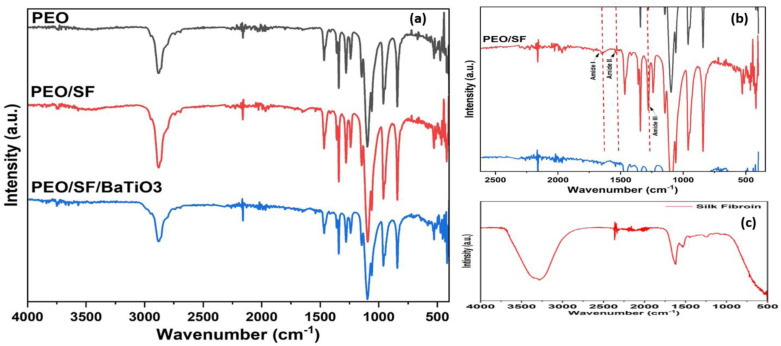
(**a**) FTIR of pristine PEO, PEO/15 wt.% SF, and PEO/15 wt.% SF/0.2 wt.% BaTiO_3_, (**b**) inset in the range of 500 to 2500 cm^−1^ for the PEO/15 wt.% SF, (**c**) FTIR for pure SF.

FTIR analysis was used to investigate the structural changes produced by mixing and stirring on the PEO/15 wt.% SF/0.2 wt.% BaTiO_3_ electrospun membranes. Figure 7 shows: (a) FTIR of pristine PEO, PEO/15 wt.% SF, and PEO/15 wt.% SF/0.2 wt.% BaTiO_3_; (b) inset in the range of 500 to 2500 cm^−1^ for the PEO/15 wt.% SF; and (c) FTIR for pure SF. As can be seen, the spectra profile corresponds to each mat. The ranges of all the tissue mats showed identical characteristic regions. The three major characteristic bands of PEO were identified through the profile zone (500–4000 cm^−1^) analysis (Figure 7a). The main conformations of SF are random coil, α-helix, and β-sheet, which is the most stable form [53]. Although the presence of ethanol as a solvent is significant in facilitating the complete solution of both PEO and SF, and comparing to FTIR of the pure SF profile Figure 7c, it induces silk molecules to form a ß-sheet structure (Figure 7b).

Moreover, the PEO/SF nanofibrous mats became hydrophobic via ethanol vapor treatment, which enhances SF conformational transition from the random coil (silk I) to β-sheet (silk II) [53]. It seems that a fraction of the random coil (silk I) turned into a β-sheet (silk II), and so PEO/SF nanofibrous matrices became hydrophobic [53]. The PEO/SF nanofibrous matrix has characteristic absorption bands of amide I (C–O stretching vibration) at (1700–1600 cm^−1^) and amide II (N–H bending vibration and C–N stretching vibration) at (1600–1500 cm^−1^) bands, amide II, and amide III (C–N stretching vibration), respectively, which represent SF [53]. Furthermore, the absorption bands around 1100 cm^−1^ correspond to C-O-C stretching vibration, which represents SF. In addition, PEO has characteristic absorption bands (C–O–C symmetric stretch vibration) at 1101 cm^−1^ [53]. C-H group peaks were observed at 1340 and 1370 cm^−1^. The broad bond showed at 2900 cm^−1^ due to the CH_2_ stretching groups. Due to the presence of ethanol as a solvent with a low content (20%), the SF characteristic absorption peaks mentioned earlier shifted to 1242 cm^−1^ and 1526 cm^−1^, respectively, suggesting a conformation change from the random coil (silk I) to a β-sheet (silk II).

**Figure 8:**
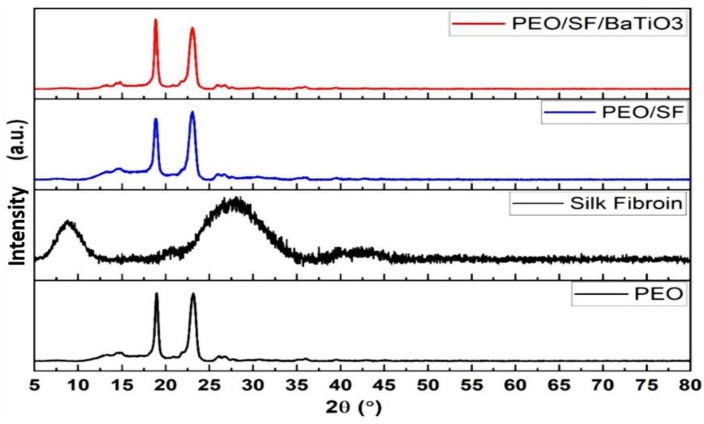
XRD of pristine PEO, PEO/15 wt.% SF, and PEO/15 wt.% SF/0.2 wt.% BaTiO_3_.

As proved in the XRD pattern of the PEO, PEO/15 wt.% SF, and PEO/15 wt.% SF/0.2 wt.% BaTiO_3_ composites (Figure 8), the diffractogram demonstrated that the pure PEO polymer powder has a crystalline structure, represented by the strongly reflected peaks at 19.6° and 23.4. In contrast, the characteristic peak of the SF at 27.5° is covered under the PEO peak. These PEO peaks were also identified and analyzed by Zhang et al. [54,55]. The distinct peaks of the BaTiO_3_ particles could not be detected due to the low concentration of the piezoelectric material additives. Further, the other crystalline peaks attributed to SF could not be identified due to the amorphous structure of the silk fibroin as an amorphous molecular aggregate in fibers.

**Figure 9:**
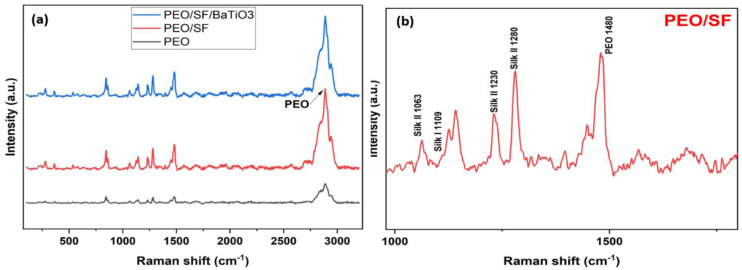
(**a**) Raman spectroscopy of pristine PEO, PEO/15 wt.% SF, and PEO/15 wt.% SF/0.2 wt.% BaTiO_3_, (**b**) inset in the range of 1000 to 2000 cm^−1^ for the PEO/15 wt.% SF.

As a non-destructive technology, Raman spectroscopy can reflect the conformational structure of molecules. Figure 9a Shows Raman spectroscopy of pristine PEO, PEO/15 wt.% SF, and PEO/15 wt.% SF/0.2 wt.% BaTiO_3_. Figure 9b shows an inset in the range of 1000 to 2000 cm^−1^ for the PEO/15 wt.% SF. This figure compares the average spectra of the blend of PEO, PEO/SF, and PEO/SF/BaTiO_3_ scaffolds. These results demonstrate that adding BaTiO_3_ particles increased the intensity of the peaks. FTIR and Raman spectroscopy confirm that, by adding more BaTiO_3_ particles, the polar phase fluctuated, increasing and then decreasing. As can be noted in Figure 9b, the peaks at 1063, 1234, 1230, and 1280 cm^−1^ arise from the silk II (β sheet conformation), and the weak absorption at 1109 cm^−1^ implies the existence of a small amount of silk I (random coil or a helical conformation). The peak at 1480 cm^−1^ belongs to PEO.

### 3.3. Piezoelectric Properties Evaluation

**Figure 10:**
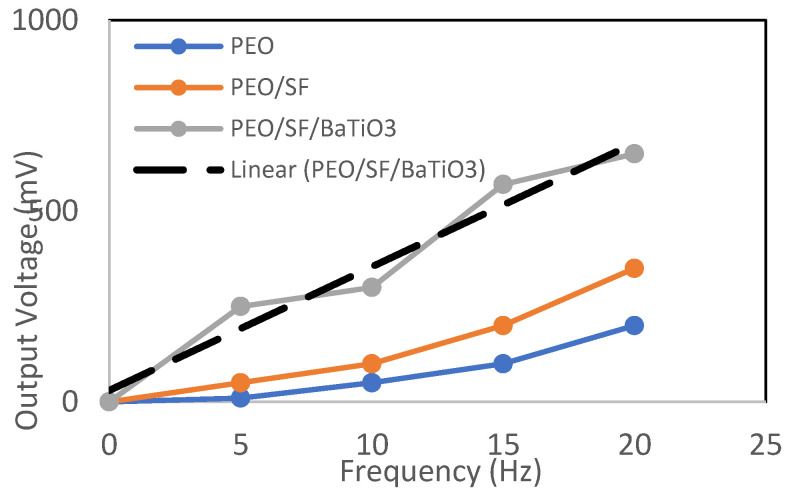
Correlation between the frequency and the output voltage for PEO, PEO/15 wt.% SF, and PEO/15 wt.% SF/0.2 wt.% BaTiO_3_ at an applied force of 5 N.

Piezoelectric impact is the capability of certain materials to produce an electric charge in response to applied mechanical pressure. Some polymer materials, such as polyvinylidene fluoride, collagen, and a combination of these with other materials such as Polycaprolactone and MWCNT, are becoming a more relevant area of research for piezoelectric biomaterials [56,57]. PVDF also has the highest piezoelectric properties, especially when mixed with BaTiO_3_-Ag. However, most of those materials are not biodegradable. SF demonstrated a high apparent piezoelectric coefficient and maintained its good electroactive properties [58]. However, the transformation from the silk I to silk II phase limits the dipole alignment to some extent due to the presence of ethanol as a solvent. Thus, adding piezoelectric nanoparticles may enhance the output voltage and piezoelectrical properties of the newly designed PEO/SF composite. Figure 10 shows the correlation between the load frequency and the output voltage for PEO, PEO/SF, and PEO/SF/BaTiO_3_. The samples produced were assessed in tapping mode, and all samples were subjected to the same applied force of 5 N and different load frequencies ranging from 5 to 20 cycles/second. It can be seen that the output voltage increases with increasing load frequency. The slope is somewhat nonlinear, possibly due to a fracture or deformation due to high load frequency. A maximum of around 700 mV has been obtained at 20 Hz frequency, and 5 N applied voltage for the designed biocomposites, which is much higher than a similar system (PVDF-BaTiO_3_-Ag fibers) reported in the literature.

**Figure 11:**
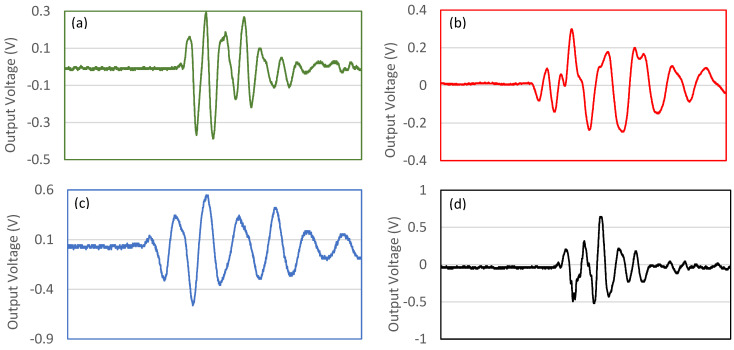
The generated harmonics noise voltage produced by the piezoelectric materials at a different frequency and 5 N applied force: (**a**) 5 Hz, (**b**) 10 Hz, (**c**) 15 Hz, and (**d**) 20 Hz for PEO/15 wt.% SF/0.2 wt.% BaTiO_3_ nanofiber mat.

Figure 11 shows samples of the generated harmonics noise voltage produced by the piezoelectric materials at a different frequency and 5 N applied force for the PEO/15 wt.% SF/0.2 wt.% BaTiO_3_ nanofiber mat. The presence of BaTiO_3_ enhanced the piezoelectric performance of the biocomposite nanofiber mat.

**Figure 12:**
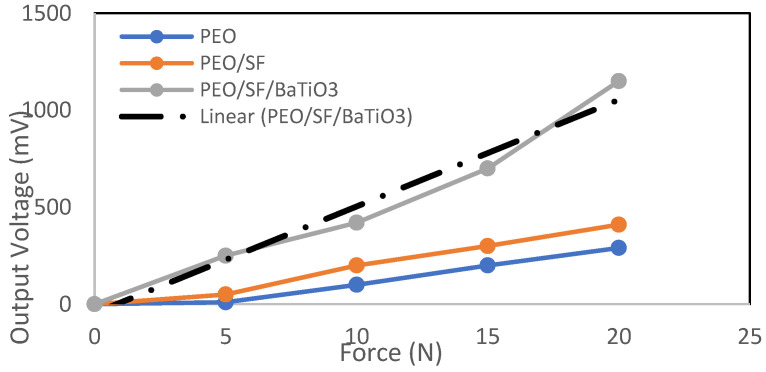
Correlation between the force and the output voltage for PEO, PEO/15 wt.% SF, and PEO/15 wt.% SF/0.2 wt.% BaTiO_3_ at a constant frequency of 5 Hz.

Figure 12 shows the correlation between the force and the output voltage for PEO, PEO/SF, and PEO/SF/BaTiO_3_ at a frequency of 5 Hz. The samples were evaluated in tapping mode, and all pieces were induced by the same cyclic load of 5 Hz frequency and different applied forces ranging from 5 to 20 N. It can be seen that the output voltage increases with increasing applied force. The slope is somewhat linear, which may be due to the low applied frequency used. Furthermore, the presence of BaTiO_3_ enhanced the piezoelectric performance of the biocomposite nanofiber mat. An output voltage of around 1150 mV was obtained at a high applied force. Compared to a similar system of PVDF-BaTiO_3_-Ag fibers (1.78(12) mV), the proposed new composites showed a better piezoelectric performance.

**Figure 13:**
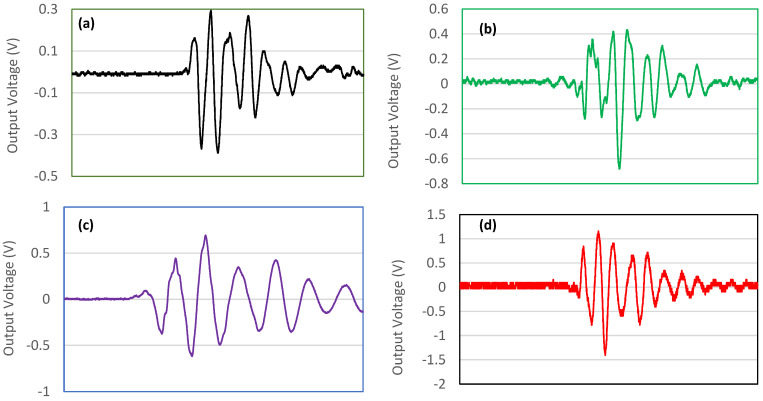
The generated harmonics noise voltage produced by the piezoelectric materials at 5 Hz frequency and different applied forces: (**a**) 5 N, (**b**) 10 N, (**c**) 15 N, and (**d**) 20 N for PEO/15 wt.% SF/0.2 wt.% BaTiO_3_ nanofibers mat.

The generated harmonics noise voltage produced by the piezoelectric materials can be seen in Figure 13. As seen in this Figure, the signal shows the electrical voltage output. The waveform of the signals is fundamental in analyzing the piezoelectric responses of each sample. As can be seen, after tapping, the oscilloscope senses a damping signal that belongs to the output voltage. With the fixed applied load, the output of the tested samples was faster and sharper, confirming the prepared samples’ piezoelectric features.

## 4. Conclusions

This work aimed to develop blends of polyethylene oxide (PEO) and silk fibroin for possible application in cardiac tissue engineering (CTE). An electrical integration function to create composite scaffolds by loading piezoelectric materials during preparation was achieved. The following points can be concluded so far:

○ Different weight percentages of SF solution were added to the PEO solution to form a PEO/SF blend with uniform nanofibers. An optimum amount of 15 wt.% of SF that produces nanofibers with a minimum amount of beads was achieved. BaTiO_3_ piezoelectric nanoparticles were added to the PEO/15 wt.% silk fibroin blend solution, and the obtained suspensions were introduced to an electrospinning device.
○ Well-organized smooth nanofibers with low bead content were obtained in the PEO/15 wt.% SF/0.2 wt.% BaTiO_3_ composite.
○ Piezoelectric characteristics were assessed by the piezotester mechanism, designed and manufactured in our lab.
○ Electrospun PEO/15 wt.% SF/0.2 wt.% BaTiO_3_ fibers increased the output voltage (1150 mV) compared to pristine PEO and PEO/SF composite fibers (410 and 290 mV, respectively) upon applying 20 N force at 5 Hz frequency, which is much higher than PVDF-BaTiO_3_-Ag fibers that were reported in the literature.

## Data Availability

Not applicable.

## Supplementary Materials

The following supporting information can be downloaded at: https://www.mdpi.com/article/10.3390/polym14194209/s1, Figure S1: SolidWorks Drawing of the PizoTester; Video S1: PiezoTester mechanism.
