# Supplementary material for: Development of New Bio-Composite of PEO/Silk Fibroin Blends Loaded with Piezoelectric Material
Source: Polymers (Basel). 2022 Oct 7;14(19):4209. doi: 10.3390/polym14194209 (PMC9571570; doi:10.3390/polym14194209)
Supplement: Supplementary file 1 [file polymers-14-04209-s001.zip › polymers-1913024-supplementary/Figure S1 SolidWork Drawing of the PiezoTester.pdf]

2 | 1

B

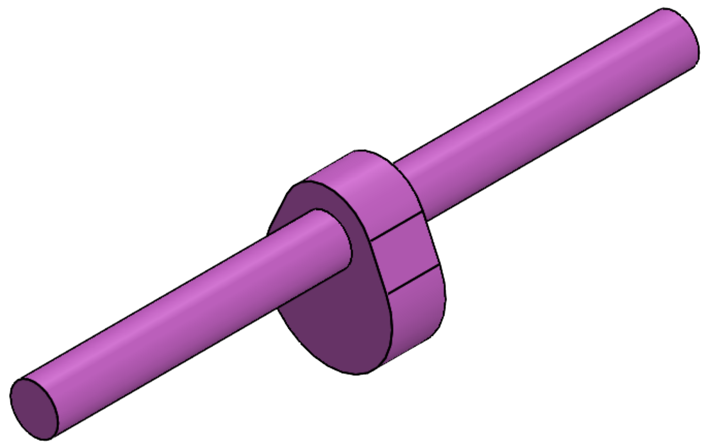

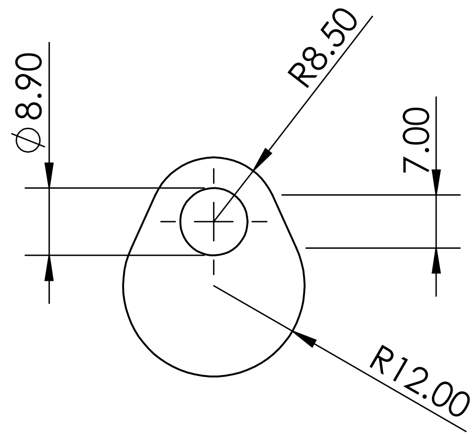

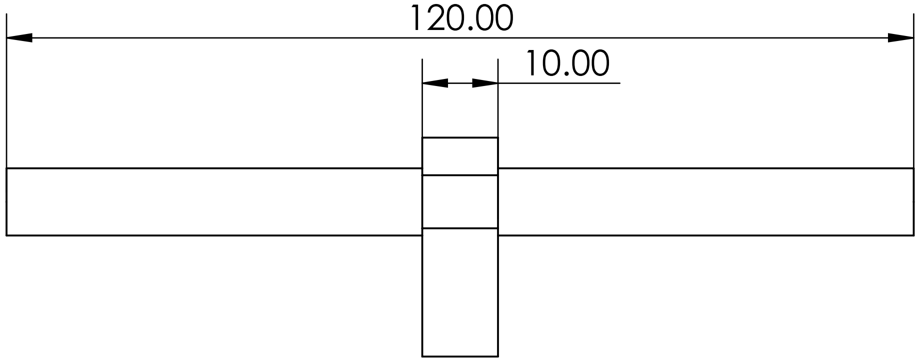

B

A

PROPRIETARY AND CONFIDENTIAL

THE INFORMATION CONTAINED IN THIS DRAWING IS THE SOLE PROPERTY OF <INSERT COMPANY NAME HERE>. ANY REPRODUCTION IN PART OR AS A WHOLE WITHOUT THE WRITTEN PERMISSION OF <INSERT COMPANY NAME HERE> IS PROHIBITED.

NEXT ASSY	USED ON
APPLICATION	

UNLESS OTHERWISE SPECIFIED:

DIMENSIONS ARE IN INCHES
TOLERANCES:
FRACTIONAL ±
ANGULAR: MACH ± BEND ±
TWO PLACE DECIMAL ±
THREE PLACE DECIMAL ±

INTERPRET GEOMETRIC TOLERANCING PER:

MATERIAL

FINISH

DO NOT SCALE DRAWING

	NAME	DATE
DRAWN		
CHECKED		
ENG APPR.		
MFG APPR.		
Q.A.		

COMMENTS:

TITLE:

SIZE A | DWG. NO. Cam and shaft | REV

SCALE: 1:2 | WEIGHT: | SHEET 1 OF 1

A

2 | 1

SOLIDWORKS Educational Product. For Instructional Use Only.

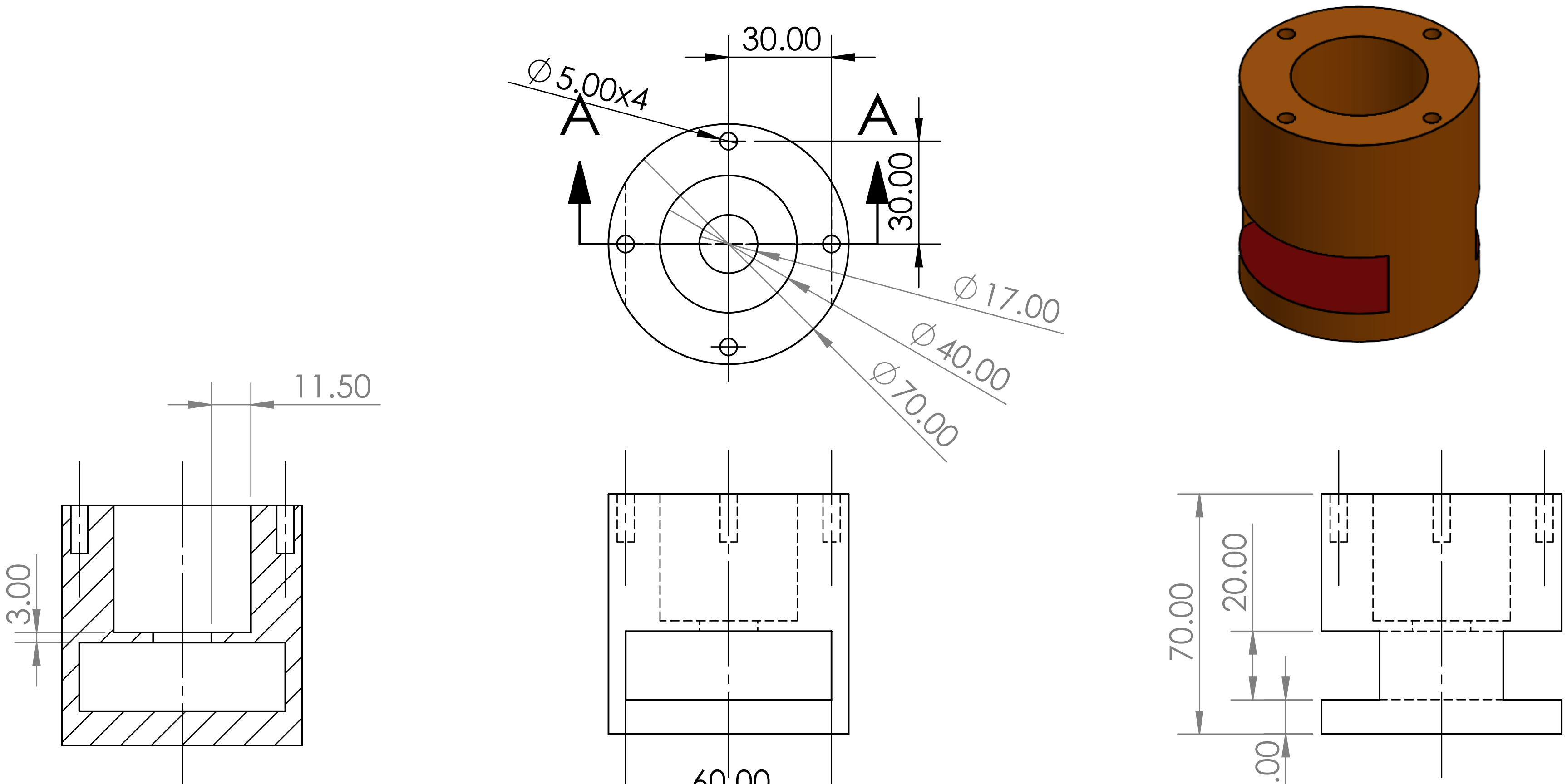

SECTION A-A

		UNLESS OTHERWISE SPECIFIED:		NAME	DATE	
		DIMENSIONS ARE IN INCHES<br>TOLERANCES:<br>FRACTIONAL ±<br>ANGULAR: MACH ± BEND ±<br>TWO PLACE DECIMAL ±<br>THREE PLACE DECIMAL ±	DRAWN			TITLE:
			CHECKED			
			ENG APPR.			
			MFG APPR.			
		INTERPRET GEOMETRIC TOLERANCING PER:	Q.A.			
		MATERIAL	COMMENTS:			SIZE **A** DWG. NO. Case REV
NEXT ASSY	USED ON	FINISH				
APPLICATION		DO NOT SCALE DRAWING				SCALE: 1:2 WEIGHT: SHEET 1 OF 1

PROPRIETARY AND CONFIDENTIAL

THE INFORMATION CONTAINED IN THIS DRAWING IS THE SOLE PROPERTY OF <INSERT COMPANY NAME HERE>. ANY REPRODUCTION IN PART OR AS A WHOLE WITHOUT THE WRITTEN PERMISSION OF <INSERT COMPANY NAME HERE> IS PROHIBITED.

SOLIDWORKS Educational Product. For Instructional Use Only.

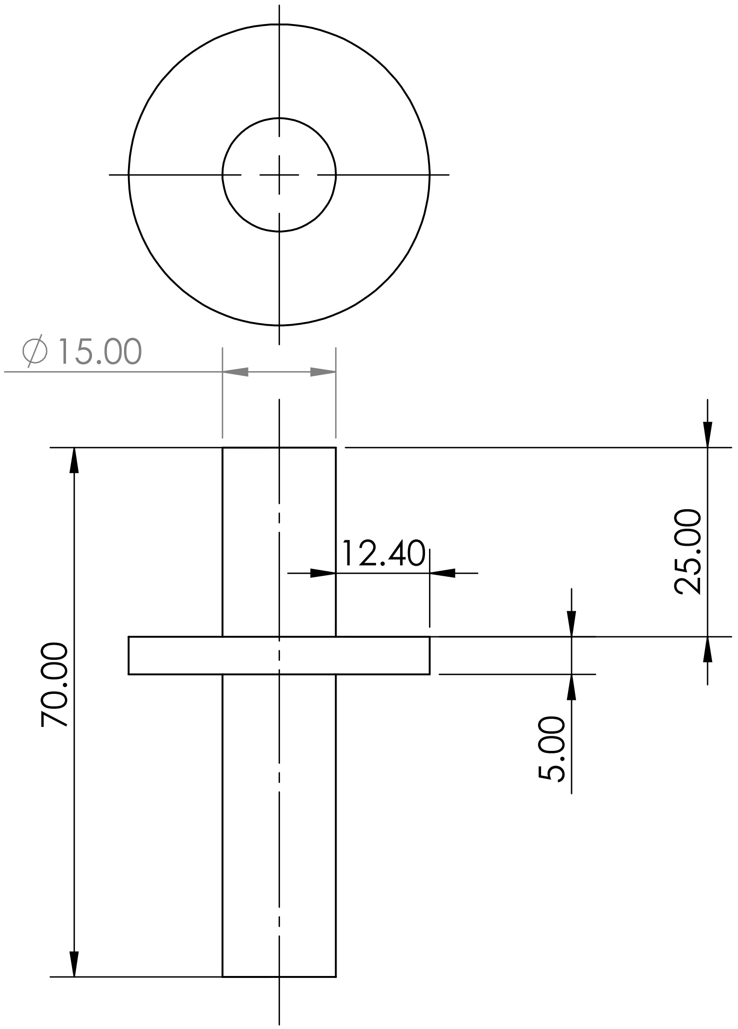

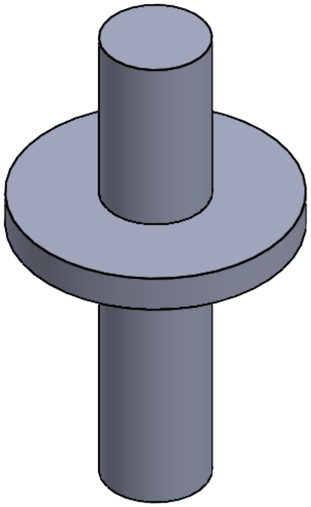

		UNLESS OTHERWISE SPECIFIED:		NAME	DATE
		DIMENSIONS ARE IN INCHES<br>TOLERANCES:<br>FRACTIONAL ±<br>ANGULAR: MACH ± BEND ±<br>TWO PLACE DECIMAL ±<br>THREE PLACE DECIMAL ±	DRAWN		
			CHECKED		
			ENG APPR.		
			MFG APPR.		
		INTERPRET GEOMETRIC TOLERANCING PER:	Q.A.		
		MATERIAL	COMMENTS:		
NEXT ASSY	USED ON	FINISH			
APPLICATION		DO NOT SCALE DRAWING			

TITLE:

SIZE **A** DWG. NO. follower REV

SCALE: 1:1 WEIGHT: SHEET 1 OF 1

PROPRIETARY AND CONFIDENTIAL

THE INFORMATION CONTAINED IN THIS DRAWING IS THE SOLE PROPERTY OF <INSERT COMPANY NAME HERE>. ANY REPRODUCTION IN PART OR AS A WHOLE WITHOUT THE WRITTEN PERMISSION OF <INSERT COMPANY NAME HERE> IS PROHIBITED.

SOLIDWORKS Educational Product. For Instructional Use Only.

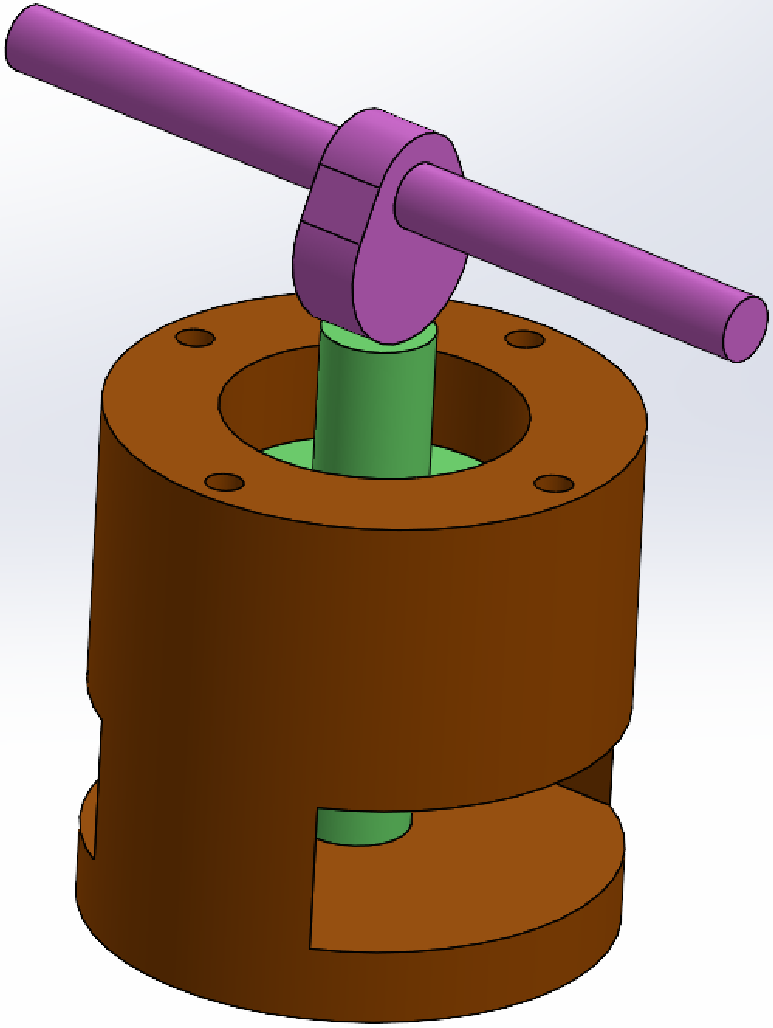

SOLIDWORKS Educational Product. For Instructional Use Only.

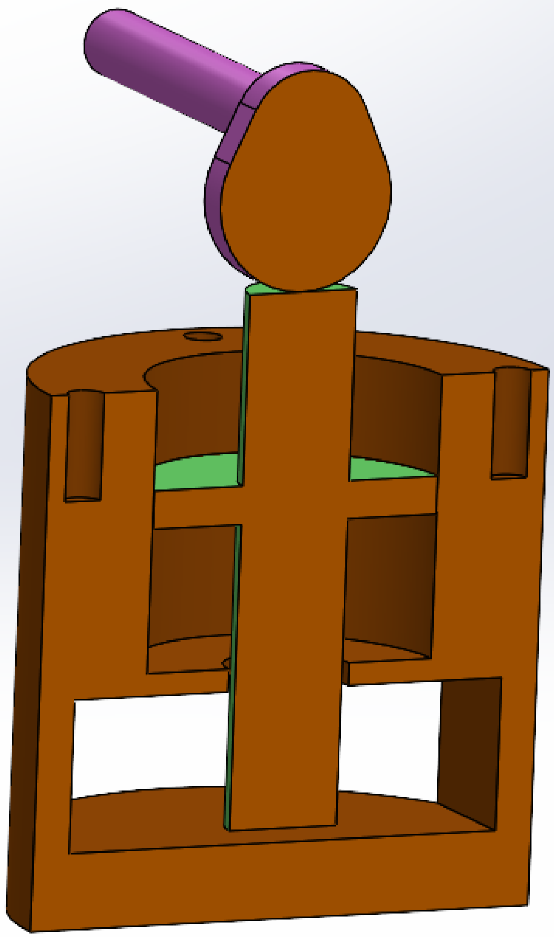

SOLIDWORKS Educational Product. For Instructional Use Only.
